# PCOS discussions on a Turkish online forum: a qualitative netnographic study of concerns, peer advice, and information gaps

**DOI:** 10.3389/frph.2026.1978089

**Published:** 2026-10-16

**Authors:** Ömercan Aksoy

**Affiliations:** Department of Midwifery, Faculty of Health Sciences, Istanbul Topkapi University, Istanbul, Türkiye

**Keywords:** fertility anxiety, misinformation, netnography, online forum, peer advice, polycystic ovary syndrome, polyendocrine metabolic ovarian syndrome

## Abstract

**Introduction:**

Polycystic ovary syndrome (PCOS) is a multidimensional endocrine, metabolic, and reproductive condition, yet little is known about how related concerns and peer advice unfold within Turkish online forums. This study examined initial concerns and associated peer-response patterns in a publicly accessible Turkish forum.

**Methods:**

A qualitative netnographic study was conducted using 120 initial posts and 708 peer responses published between August 2025 and May 2026. Initial posts were analyzed using reflexive thematic analysis and assigned one dominant theme for descriptive frequency analysis. Peer responses were categorized by their dominant communicative function using qualitative content analysis.

**Results:**

Fertility, pregnancy, and motherhood anxiety was the most frequent initial concern (*n* = 24, 20.0%), followed by treatment and medication-related uncertainty (*n* = 22, 18.3%). Among peer responses, incomplete or uncertain advice predominated (*n* = 398, 56.2%), followed by biomedical guidance or normalization (*n* = 137, 19.4%), practical navigation (*n* = 50, 7.1%), and experiential advice (*n* = 47, 6.6%). Cross-tabulation demonstrated that incomplete or uncertain advice predominated across most initial concern categories.

**Discussion:**

Turkish PCOS forum discussions represent a mixed peer-information environment in which reassurance, experiential knowledge, practical guidance, incomplete advice, non-evidence-based recommendations, and stigmatizing interactions coexist. Healthcare professionals should address patients' online information-seeking and provide clear, person-centered guidance on diagnosis, treatment, fertility, visible symptoms, and healthcare pathways.

## 1 Introduction

Polycystic ovary syndrome is one of the most common endocrine, metabolic, and reproductive disorders affecting women and people assigned female at birth during the reproductive years. A recent global systematic review and meta-analysis estimated the worldwide prevalence of polycystic ovary syndrome as 9.2%, although estimates vary according to diagnostic criteria, population characteristics, and study methodology (1). The syndrome is clinically heterogeneous and may involve ovulatory dysfunction, hyperandrogenism, polycystic ovarian morphology, metabolic disturbances, dermatological manifestations, and psychological concerns (2). The 2023 International Evidence-based Guideline for the Assessment and Management of Polycystic Ovary Syndrome emphasizes that care should be person-centered and should address reproductive, metabolic, dermatological, psychological, and lifestyle-related dimensions of the condition (2). In May 2026, an international multistakeholder consensus process renamed the condition polyendocrine metabolic ovarian syndrome (PMOS) to better reflect its multisystem endocrine, metabolic, and ovarian features (3). Because the forum corpus and search terminology in the present study were collected using the established PCOS terminology, PCOS is retained when describing the source material and search strategy, while the updated PMOS terminology is acknowledged here.

Although polycystic ovary syndrome is often introduced clinically through menstrual irregularity, hyperandrogenism, infertility, and metabolic risk, its consequences extend beyond biomedical symptoms. Individuals with the syndrome may experience acne, hirsutism, hair loss, weight-related concerns, body dissatisfaction, fertility-related fears, anxiety, depressive symptoms, eating-related concerns, and reduced health-related quality of life (2, 4–6). Previous research has shown that polycystic ovary syndrome can affect quality of life through concerns related to hirsutism, menstruation, emotions, infertility, weight, and body image (5, 6). Current clinical recommendations also emphasize the need to assess and address mental health symptoms, body image distress, eating disorders, psychosexual function, and quality of life as part of polycystic ovary syndrome care (2, 4). These findings indicate that polycystic ovary syndrome should be understood not only as a reproductive-endocrine disorder but also as a condition that shapes self-perception, emotional well-being, social participation, and reproductive expectations.

Despite advances in clinical guidelines, people with polycystic ovary syndrome continue to report challenges related to diagnosis, information provision, follow-up care, and disease-management support (7–9). Large-scale and patient-centered studies have identified delayed diagnosis, inadequate information, dissatisfaction with care, and unmet needs for multidisciplinary support as recurring problems in polycystic ovary syndrome care (7, 9). A systematic review of lived experiences also highlighted the need for holistic care and co-created educational resources for people living with the condition (8). These gaps are clinically important because polycystic ovary syndrome often requires coordinated care across gynecology, endocrinology, dermatology, nutrition, mental health, and primary care (2, 7–9). When clinical explanations or care pathways are perceived as insufficient, individuals may seek additional information, validation, and practical guidance from online sources (10, 11).

Online platforms have become important spaces where people seek, interpret, and exchange health-related information. For polycystic ovary syndrome, digital environments may serve as supplementary spaces for understanding diagnosis, comparing symptoms, evaluating treatment options, and sharing lived experiences (10, 11). A recent qualitative study found that women with polycystic ovary syndrome used online health resources and social media to make sense of a diagnosis they perceived as complex and poorly understood (10). A cross-sectional analysis of polycystic ovary syndrome-related content across TikTok, Instagram, and Reddit also showed that online discussions commonly cover symptoms, interventions, interactions with the medical system, and information-seeking (11). These studies suggest that online platforms may influence how individuals understand symptoms, treatments, fertility, and interactions with healthcare professionals.

Online health spaces may provide emotional validation, peer support, experiential knowledge, and practical navigation of healthcare services (10, 11). However, they may also expose users to incomplete, misleading, non-evidence-based, or stigmatizing information (11–14). Polycystic ovary syndrome-related online content is particularly relevant to this concern because users often search for advice about hormonal medications, contraception, fertility treatments, dermatological symptoms, weight management, diet, supplements, herbal remedies, and self-directed strategies (2, 10, 13, 14). Studies of polycystic ovary syndrome-related online content have raised concerns about the quality, reliability, and accuracy of information, including misinformation around symptoms, causes, treatments, and alternative approaches (11, 13, 14). More broadly, health misinformation in digital environments can influence beliefs, risk perceptions, care-seeking decisions, and health behaviors (12).

Although international research has begun to examine PCOS-related social media and online health information, less is known about how the condition is discussed in Turkish online forums. A Turkish dataset is relevant because the meanings attached to fertility, visible symptoms, disclosure, family expectations, and healthcare navigation are shaped by local social and health-system contexts. Turkish studies have documented substantial quality-of-life and body-image burdens associated with hirsutism and PCOS-related symptoms (15, 16), but naturally occurring Turkish peer-to-peer forum exchanges remain underexplored. Forum-based analysis is particularly useful because initial questions can be examined together with the responses they elicit, allowing concern-specific patterns of reassurance, experiential advice, practical navigation, incomplete guidance, alternative recommendations, and stigmatizing interactions to be described.

This study aimed to map polycystic ovary syndrome-related discussions in a Turkish online forum by analyzing both initial posts and associated peer responses.

## 2 Materials and methods

### 2.1 Study design

This study was designed as a qualitative netnographic study with descriptive content analysis. Netnography is suitable for examining naturally occurring interactions within online communities and digital social spaces (17). In this study, a Turkish online forum was treated as a digital health-related space where users discussed polycystic ovary syndrome, shared concerns, and responded to one another's questions.

The study included two linked analytic components. Initial forum posts were analyzed using reflexive thematic analysis to identify users' information needs, lived concerns, and help-seeking patterns related to polycystic ovary syndrome (18). Associated peer responses were analyzed using qualitative content analysis to identify response patterns, including biomedical guidance, emotional support, experiential advice, practical navigation, alternative advice, incomplete advice, and stigmatizing or inappropriate responses (19, 20).

### 2.2 Research questions

This study was guided by the following research questions:

What dominant thematic concerns are expressed in initial forum posts about polycystic ovary syndrome, and how are these concerns distributed descriptively within the dataset?

What dominant peer response patterns are observed in responses to polycystic ovary syndrome-related initial posts, and how are these patterns distributed descriptively within the dataset?

How do peer-response patterns vary across the dominant thematic concerns expressed in initial posts?

### 2.3 Data source and sampling

Data were collected from KızlarSoruyor, a publicly accessible Turkish online question-and-answer forum. The platform allows users to post questions and receive responses on topics including health, relationships, body image, sexuality, and family life. Only publicly visible content was used. The researchers did not contact users, access private messages, or collect non-public information. The dataset covered forum posts published between August 2025 and May 2026. Within this predefined period, all publicly accessible Turkish forum threads/posts containing the keyword ‘PCOS’, ‘polikistik over’ and ‘polikistik over sendromu’, and meeting the inclusion criteria were retrieved; no additional purposive or theoretical sampling was applied.

Forum threads were identified using the prespecified keywords. Threads were eligible when the initial post or discussion explicitly referred to PCOS, was written in Turkish, was publicly accessible, and contained a question, concern, experience, or help-seeking statement related to the condition. Duplicate/reposted material, content not primarily related to PCOS, platform/interface material, and entries without analyzable user-generated content were excluded. Because the platform workflow did not preserve a machine-readable count of all search-result hits, an exact retrospective count of records identified before eligibility screening could not be verified and is not reconstructed here. The final eligible initial-post dataset comprised 120 posts. Figure 1 reports all verifiable counts from the response-level re-audit.

**Figure 1:**
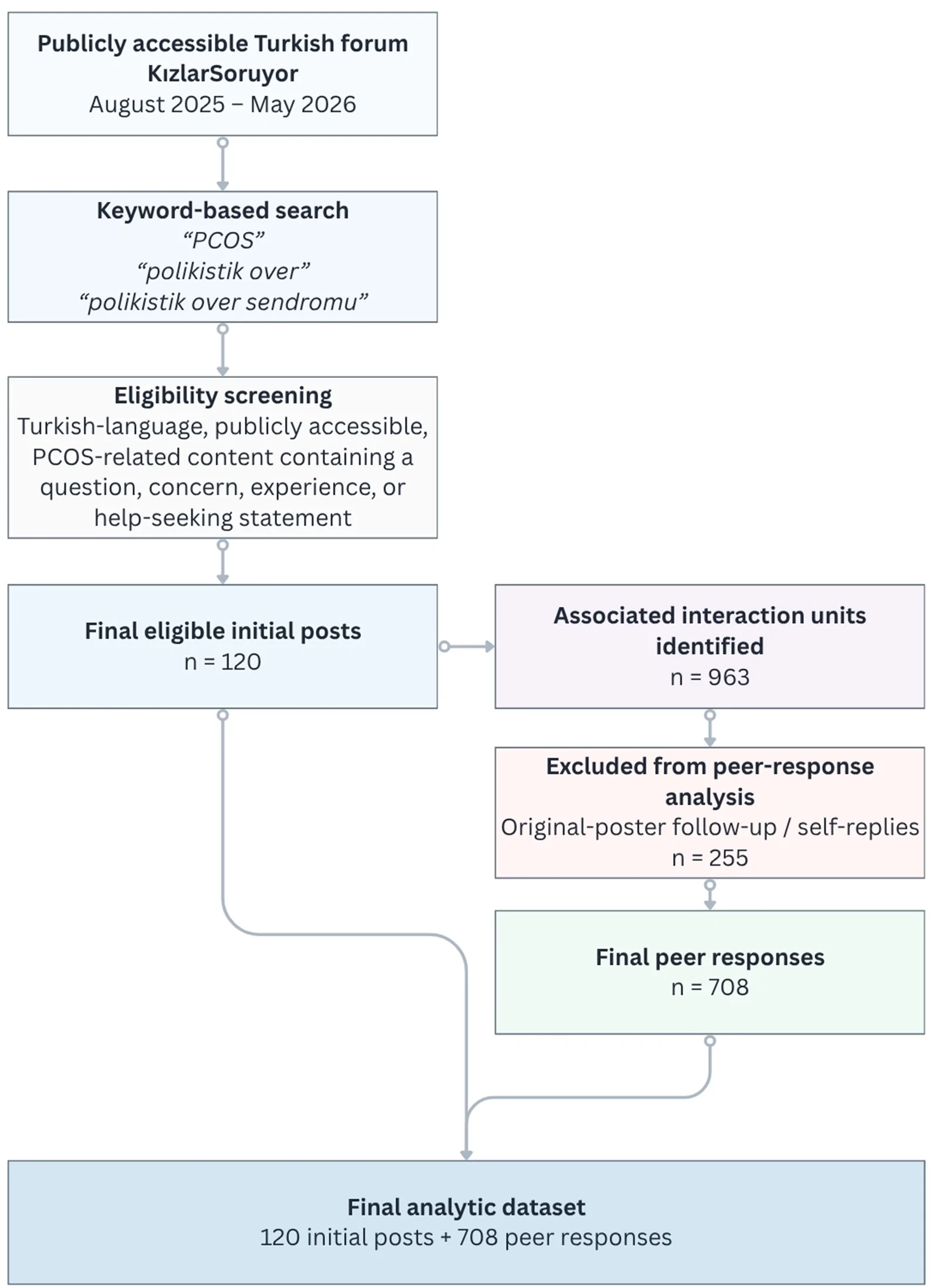
Identification, eligibility, and response-level re-audit of PCOS-related forum material. The final initial-post dataset comprised 120 eligible posts. During revision-stage response-level re-audit, 963 time-stamped interaction units were identified; 255 original-poster follow-up/self-replies were excluded, leaving 708 peer responses. The final analytic corpus therefore contained 828 units (120 initial posts+708 peer responses).

During revision, the raw forum corpus was re-audited after the peer-response coding framework was operationalized more explicitly. The re-audit identified 963 time-stamped interaction units associated with the corpus. Follow-up comments attributable to the original thread authors were not treated as peer responses; 255 such original-poster/self-reply units were excluded. Short but meaningful responses from other users were retained regardless of length. The final response-level analytic dataset therefore comprised 708 peer responses. Initial posts and peer responses were treated as linked but analytically distinct units, yielding 828 analytic units in total (120 initial posts+708 peer responses).

### 2.4 Data preparation and ethical handling

The copied forum material was cleaned before analysis. Usernames, profile identifiers, platform badges, scores, links, exact dates, and interface elements were removed. Where posts contained location, hospital, physician, or highly specific personal details, these were generalized or excluded from reporting. Visible age information was recorded only when displayed in the forum profile or post metadata; because such information was self-reported and unverifiable, it was used only for descriptive contextualization.

Each forum thread was assigned an anonymous thread identifier. Initial posts were labeled as T01-P, T02-P, and so forth, while associated responses were labeled according to their thread and response order, such as T01-R1 or T01-R2. These identifiers were created by the researchers and do not correspond to usernames, profile names, or platform identifiers. They were used only to link analytic examples to an initial post or a response within the same thread without revealing platform identities.

Although the data were publicly accessible, PCOS-related posts were treated as sensitive health-related narratives because they included information about menstruation, infertility, medication use, body image, mental health, sexuality, and family relationships. Ethical decisions were informed by the Association of Internet Researchers guidelines, which emphasize contextual privacy, potential harm, and traceability in internet-mediated research (21). No verbatim forum text is reported in the manuscript. Illustrative content is presented only as researcher-generated, non-verbatim summaries of the meaning of posts and responses. Usernames, URLs, exact dates, and directly identifying details are not reported, and highly specific contextual details were generalized when necessary to reduce traceability.

### 2.5 Data analysis

Data analysis proceeded in two stages. First, initial posts were analyzed using reflexive thematic analysis (18). Posts were read repeatedly in Turkish, initial codes were generated, and codes were grouped into candidate themes. Themes were then reviewed, refined, named, and interpreted in relation to the study aim. Each initial post was assigned one dominant theme for descriptive frequency analysis. Although some posts contained multiple concerns, assigning one dominant theme enabled the analysis to show the relative prominence of concerns across the dataset. The dominant theme was determined according to the main question, concern, or help-seeking purpose expressed in the initial post.

Second, associated responses were analyzed using qualitative content analysis (19, 20). Each peer response was assigned one dominant response pattern according to its primary communicative function in context. The seven operational categories were defined as follows: (1) biomedical guidance/normalization: a biomedical explanation, normalization, or clinically oriented recommendation when this was the response's dominant function; (2) incomplete/uncertain advice: brief, vague, unsupported, uncertain, or minimally explanatory guidance; (3) alternative/non-evidence-based advice: herbal, supplement-based, ‘natural,’ or other non-evidence-based approaches presented as management; (4) emotional support/validation: empathy, acknowledgment of distress, reassurance, or psychosocial support as the dominant function; (5) dismissive/stigmatizing/inappropriate responses: minimization, mocking, sexualization, shaming, or stigmatizing content; (6) experiential advice: personal or observed experience used as guidance; and (7) practical navigation: concrete guidance about healthcare services, specialties, hospitals, appointments, or system navigation. When a response contained more than one function, the category representing the dominant communicative function in the full thread context was assigned. Short responses were retained when they represented genuine peer interaction rather than being excluded solely because of length.

OpenAI ChatGPT was used as an assistive analytic tool for data organization, preliminary coding, structured recoding, and language editing. The initial AI-assisted workflow used ChatGPT (GPT-5.5 Thinking), and the revision-stage raw-corpus audit and recoding used ChatGPT (GPT-5.6 Sol). Prompts instructed the model to organize text into candidate codes, apply the predefined operational response categories, flag ambiguous entries, and return structured outputs for researcher review; the model was not asked to make autonomous clinical judgments. Publicly accessible forum text was processed within the ChatGPT environment as part of this workflow; no private messages or non-public platform data were collected. Because the corpus contained sensitive health narratives, this AI-assisted processing is explicitly acknowledged as a methodological and privacy consideration. The AI-assisted recoding was not an independent second coding by a human researcher and was not manually re-coded response-by-response by the author. Researcher oversight focused on the coding framework, flagged or ambiguous cases, consistency checks, summary distributions, and interpretation of the findings; final interpretive responsibility remained with the author.

Descriptive frequencies and percentages were calculated for both dominant themes in initial posts and dominant peer-response patterns. To address the relationship between concerns and the responses they elicited, each of the 708 peer responses was linked to the dominant theme of its corresponding initial post and a theme-by-response-pattern cross-tabulation was generated. Percentages in this cross-tabulation were calculated within each initial-post theme. Counts were used descriptively to indicate relative prominence and were not intended for inferential statistical comparison (22). The adequacy of the qualitative corpus was considered in relation to information power and thematic sufficiency rather than statistical power calculation (23, 24).

### 2.6 Trustworthiness and reporting

Trustworthiness was supported through an audit trail linking the raw forum corpus, cleaning decisions, operational category definitions, AI-assisted provisional coding, researcher oversight, and final descriptive tables. The revision-stage re-audit specifically distinguished original-poster follow-up comments from peer responses and retained short but meaningful peer interactions. Flagged, ambiguous, or multifunctional entries were reviewed in context using the dominant-communicative-function rule. Researcher-generated, non-verbatim summaries were used to illustrate themes and response patterns without reproducing searchable forum wording. Dependability was further supported by documenting changes made to the coding framework during revision and by checking the concern-by-response cross-tabulation against stronger thread-linkage subsets before final interpretation.

The reporting of this study was informed by the Standards for Reporting Qualitative Research, which provide recommendations for transparent reporting of qualitative research design, context, sampling, data collection, data analysis, ethical considerations, and findings (25).

## 3 Results

### 3.1 Dataset overview

The final analytic dataset consisted of 120 initial posts related to PCOS and 708 peer responses. The response-level re-audit identified 963 time-stamped interaction units; 255 follow-up/self-replies attributable to original posters were excluded from peer-response analysis. Initial posts were analyzed to identify users' information needs, lived concerns, and help-seeking patterns, whereas peer responses were analyzed to characterize how other forum users responded to these concerns.

Visible age information was available for most initial posters. Most users who initiated posts were aged 18–24 years, followed by those aged 25–29 years. Because age information was self-reported in visible profile data and could not be verified, it was used only for descriptive contextualization and not treated as a clinically verified characteristic.

### 3.2 Dominant themes in initial posts

Table 1 summarizes the dominant themes identified in the initial posts. Fertility, pregnancy, and motherhood anxiety was the most frequent concern (*n* = 24, 20.0%), followed by treatment and medication-related uncertainty (*n* = 22, 18.3%). Diagnostic uncertainty and unmet information needs (*n* = 18, 15.0%) and visible symptoms, body image, and embodied distress (*n* = 18, 15.0%) were also common. Together, the five most frequent themes accounted for 81.6% of all initial posts.

**Table 1:** Distribution of dominant themes in initial polycystic ovary syndrome-related posts.

Dominant theme	*n*	%	Illustrative non-verbatim summary
Fertility, pregnancy, and motherhood anxiety	24	20.0	Strong desire for motherhood accompanied by uncertainty about future pregnancy.
Treatment and medication-related uncertainty	22	18.3	Persistent acne and hair growth despite normal blood tests, with uncertainty about treatment options.
Diagnostic uncertainty and unmet information needs	18	15.0	Uncertainty about whether hormone testing should be timed with menstruation.
Visible symptoms, body image, and embodied distress	18	15.0	Regular periods alongside distress from acne, oily skin, hair growth, and hair loss.
Nutrition, weight, and metabolic concerns	16	13.3	Being thin and unable to gain weight despite having polycystic ovary syndrome.
Disclosure dilemmas and family expectations	8	6.7	Concern that a fiancé's family might react negatively to the diagnosis.
Healthcare access and specialist navigation	7	5.8	Dissatisfaction after several medical consultations and desire for a more experienced clinician.
Psychosocial burden and coping difficulties	4	3.3	Fatigue with ongoing medication and feeling that others do not understand the burden of the condition.
Alternative/self-directed management attempts	3	2.5	Interest in whether herbal approaches might help manage polycystic ovary syndrome.
Total	120	100.0	—

#### 3.2.1 Fertility, pregnancy, and motherhood anxiety

Fertility, pregnancy, and motherhood anxiety was the most frequent theme. Users asked whether polycystic ovary syndrome would prevent pregnancy, whether conception without medication was possible, whether regular menstruation increased the likelihood of pregnancy, and whether ovulation induction treatments had worked for others. Several users expressed a strong desire for motherhood alongside uncertainty about whether polycystic ovary syndrome would allow them to conceive naturally or at all (T17-P, T43-P, T63-P). In some posts, this concern was intensified by family expectations; one user described pressure from her husband's family to have a child after approximately one year of marriage (T04-P).

#### 3.2.2 Treatment and medication-related uncertainty

The second most frequent theme involved treatment and medication-related uncertainty. Users asked which medications were commonly prescribed, whether oral contraceptives or antiandrogenic medications could be used for acne and hirsutism, whether antidepressants were compatible with the condition, and whether dermatologists or gynecologists should manage polycystic ovary syndrome-related acne. Treatment uncertainty was particularly visible when users experienced distressing symptoms despite normal laboratory findings. For example, one user described persistent acne and excessive hair growth despite normal blood tests and wondered whether treatment would still be offered; the same thread reflected frustration that menstrual-regulating treatment did not address the symptoms that were most troubling to her (T24-P). Another user questioned whether isotretinoin would be effective for acne attributed to polycystic ovary syndrome or whether symptoms might recur (T26-P). Concerns about mental health treatment also appeared, as one user asked whether physicians would avoid prescribing antidepressants because of polycystic ovary syndrome (T02-P).

#### 3.2.3 Diagnostic uncertainty and unmet information needs

Diagnostic uncertainty and unmet information needs formed another major theme. Users frequently asked whether hormone testing had to be performed during menstruation, which day of the cycle was appropriate, how long test results would take, and how the condition could be diagnosed. Some users sought clarification about whether hormonal testing needed to coincide with menstruation or whether they should return to a clinician after receiving a diagnosis with little explanation (T01-P, T82-P). A recurring source of confusion was the mismatch between ultrasound findings and laboratory results; one user described polycystic ovarian findings on ultrasound despite normal hormone tests (T13-P). Another user questioned how the diagnosis applied to her because she was thin, had regular periods, and reported no insulin resistance (T19-P).

#### 3.2.4 Visible symptoms, body image, and embodied distress

Visible symptoms, body image, and embodied distress were central to users' narratives. Acne, oily skin, hirsutism, hair loss, abdominal fat, bloating, weight gain, and inability to gain weight were frequently discussed. Users did not present these symptoms merely as cosmetic concerns; rather, they linked them to self-confidence, social participation, femininity, and daily quality of life. One user with regular periods and no reported insulin resistance described being affected by acne, oily skin, male-pattern hair growth, hair loss, and mood changes (T19-P). Another described being very thin and feeling ashamed to wear short sleeves because people repeatedly commented on her body (T27-P). Hirsutism was also distressing; one user described facial and body hair growth, uncertainty about what to do, and inability to afford treatment (T29-P).

#### 3.2.5 Nutrition, weight, and metabolic concerns

Nutrition, weight, and metabolic concerns were also common. Users discussed difficulty losing weight, inability to gain weight, sweet or chocolate cravings, gluten-free diets, diabetes risk, liver fat accumulation, and uncertainty about how to eat with polycystic ovary syndrome. These concerns were heterogeneous. Some users described weight gain and metabolic complications, whereas others described being underweight and unable to gain weight. One user emphasized that, unlike the body-weight pattern she commonly associated with polycystic ovary syndrome, she was thin and unable to gain weight (T23-P). Another reported normal blood sugar but intense chocolate cravings (T30-P). Economic limitations also shaped dietary concerns; one student described difficulty affording a gluten-free diet recommended by her physician (T40-P). Another user reported substantial weight gain and liver fat accumulation and expressed concern about whether recovery was still possible (T21-P).

#### 3.2.6 Disclosure dilemmas and family expectations

Disclosure dilemmas and family expectations appeared less frequently but were analytically important. Some users described uncertainty about whether polycystic ovary syndrome should be disclosed to a partner, fiancé, spouse, or partner's family. The central concern was that the condition might be interpreted as infertility and could lead to rejection, relationship conflict, or family pressure. One user reported that her fiancé was accepting of the diagnosis but worried that his family might reject her because of it (T09-P). Another distinguished polycystic ovary syndrome from infertility but remained uncertain about future pregnancy and feared that disclosure could affect how she was viewed by a potential partner's family (T11-P). A further user asked whether the diagnosis should be disclosed to the family of someone she might marry (T66-P).

#### 3.2.7 Healthcare access and specialist navigation

Healthcare access and specialist navigation appeared in posts where users asked for doctor, hospital, dermatologist, gynecologist, endocrinologist, dietitian, or public hospital recommendations. Users often described dissatisfaction with previous consultations or uncertainty about which specialty should manage their symptoms. One user reported having consulted several physicians without satisfactory improvement and sought a clinician with greater relevant experience (T17-P). Another asked whether dermatology or gynecology would be more appropriate for polycystic ovary syndrome-related acne (T20-P). Financial accessibility was also visible; one user stated that she needed a public hospital because she was a student and could not afford private care (T12-P).

#### 3.2.8 Psychosocial burden and coping difficulties

Psychosocial burden and coping difficulties appeared in fewer initial posts but involved intense emotional content. Users described emotional emptiness, medication fatigue, compulsive eating, body dissatisfaction, loneliness, hopelessness, and feeling misunderstood. One user described taking several medications and increasingly using food to cope with a sense of emotional emptiness (T10-P). The same thread conveyed profound fatigue with long-term medication, feeling misunderstood, and reduced desire to leave home.

#### 3.2.9 Alternative and self-directed management attempts

A smaller but important theme involved alternative and self-directed management attempts. Users asked about herbal remedies, onion cure, herbal teas, food-based methods to induce menstruation, visiting herbalists, or using medications without sufficient professional supervision. Users asked whether herbal remedies or natural methods could help with polycystic ovary syndrome (T97-P), whether onion cure or herbal tea had been tried by others (T70-P), and whether certain fruits or foods could make menstruation start (T76-P).

### 3.3 Peer response patterns

Peer responses were coded according to their dominant communicative function. Table 2 presents the final response typology after revision-stage recoding. Incomplete or uncertain advice was the most frequent response pattern (*n* = 398, 56.2%), followed by biomedical guidance or normalization (*n* = 137, 19.4%). Practical navigation accounted for 50 responses (7.1%), experiential advice for 47 (6.6%), emotional support or validation for 39 (5.5%), alternative or non-evidence-based advice for 22 (3.1%), and dismissive, stigmatizing, or inappropriate responses for 15 (2.1%).

**Table 2:** Distribution of peer response patterns after revision-stage recoding.

Peer response pattern	*n*	%	Illustrative non-verbatim summary
Incomplete/uncertain advice	398	56.2	Brief, vague, unsupported, or minimally explanatory guidance.
Biomedical guidance/normalization	137	19.4	Biomedical explanation, normalization, or clinically oriented guidance.
Practical navigation	50	7.1	Concrete guidance about specialists, hospitals, appointments, or services.
Experiential advice	47	6.6	Personal or observed experience used as guidance.
Emotional support/validation	39	5.5	Acknowledgment of distress, empathy, or reassurance.
Alternative/non-evidence-based advice	22	3.1	Herbal, supplement-based, natural, or other non-evidence-based management suggestions.
Dismissive/stigmatizing/inappropriate responses	15	2.1	Minimization, mocking, shaming, sexualization, or stigmatizing content.
Total	708	100.0	—

#### 3.3.1 Biomedical guidance and normalization

Biomedical guidance and normalization included general explanations about pregnancy, hormone testing, medication, menstrual timing, or the need for medical evaluation. In response to fertility concerns, some respondents conveyed that polycystic ovary syndrome does not necessarily preclude pregnancy while emphasizing the importance of medical follow-up (T04-R3). In response to questions about hormone test timing, respondents commonly recommended testing around the second or third day of menstruation (T01-R1).

#### 3.3.2 Incomplete or uncertain advice

Incomplete or uncertain advice often consisted of very brief, unsupported reassurance, encouragement to try something, simple affirmative or negative answers, or statements minimizing the need for concern. For example, some responses to questions about pregnancy without medication merely encouraged the user to try, without explanation (T43-R2). Other responses offered general reassurance that the situation would probably be acceptable but did not provide supporting information (T45-R1).

#### 3.3.3 Alternative or non-evidence-based advice

Alternative or non-evidence-based advice included recommendations for herbal or natural approaches, such as onion cure, herbal teas, herbalists, or food-based methods to regulate menstruation. In response to a post asking whether conception without medication was possible, one respondent recommended an onion-based remedy (T04-R1). In another fertility-related thread, a respondent attributed improvement in ultrasound findings to herbal methods (T98-R4).

#### 3.3.4 Emotional support and validation

Emotional support and validation appeared in responses that acknowledged distress, reassured users that they were not alone, and encouraged psychological or social support. In response to a post describing emotional exhaustion and eating to cope with distress, one respondent suggested that the condition, medication burden, emotional distress, and eating behavior could reinforce one another (T10-R2). Another encouraged a user expressing hopelessness to seek out someone who would genuinely listen and provide support (T10-R4).

#### 3.3.5 Dismissive, stigmatizing, or inappropriate responses

Dismissive, stigmatizing, or inappropriate responses included comments minimizing symptoms, mocking fertility concerns, making body-related judgments, or sexualizing the discussion. In response to a post about family pressure and fertility anxiety, some users made mocking comments about having many children or being replaced by another woman (T14-R3). In response to a post about being underweight and ashamed, some responses focused judgmentally on the user's body, such as saying that she needed to gain weight immediately (T27-R1).

#### 3.3.6 Experiential advice

Experiential advice consisted of users sharing their own experiences with polycystic ovary syndrome, fertility, medication, acne, hirsutism, or weight. In response to a post asking whether natural pregnancy was possible, one respondent wrote that she also had polycystic ovary syndrome and became pregnant without medication (T43-R1). In response to a post about fertility treatment, another user shared that she became pregnant after an ovulation injection (T74-R2).

#### 3.3.7 Practical navigation

Practical navigation responses provided concrete recommendations such as hospitals, physicians, university hospitals, dermatologists, dietitians, or public services. In response to a post asking for a physician experienced in polycystic ovary syndrome and infertility, one respondent suggested trying a university hospital (T17-R2). In response to a post about acne, another user suggested that dermatology could evaluate acne while hormonal causes should also be considered (T20-R1).

### 3.4 Concern-by-response patterns

Table 3 links each peer response to the dominant theme of its corresponding initial post. Incomplete or uncertain advice predominated across most concern categories, including fertility, pregnancy, and motherhood anxiety (121/200, 60.5%), diagnostic uncertainty and unmet information needs (89/155, 57.4%), healthcare access and specialist navigation (41/76, 53.9%), and treatment and medication-related uncertainty (36/67, 53.7%). Practical navigation was relatively more common in healthcare-access discussions (11/76, 14.5%), whereas biomedical guidance or normalization represented almost one-third of responses to visible-symptom/body-image posts (20/62, 32.3%). Disclosure/family-expectation threads showed a comparatively larger share of emotional support or validation (4/24, 16.7%) and dismissive/stigmatizing/inappropriate responses (2/24, 8.3%).

**Table 3:** Cross-tabulation of initial post themes and peer response patterns.

Initial post theme	Incomplete/uncertain advice	Biomedical guidance/normalization	Practical navigation	Experiential advice	Emotional support/validation	Alternative/non-evidence-based advice	Dismissive/stigmatizing/inappropriate responses	Total
Fertility, pregnancy, and motherhood anxiety	121 (60.5%)	32 (16.0%)	12 (6.0%)	15 (7.5%)	7 (3.5%)	11 (5.5%)	2 (1.0%)	200
Treatment and medication-related uncertainty	36 (53.7%)	13 (19.4%)	4 (6.0%)	5 (7.5%)	7 (10.4%)	1 (1.5%)	1 (1.5%)	67
Diagnostic uncertainty and unmet information needs	89 (57.4%)	32 (20.6%)	10 (6.5%)	11 (7.1%)	8 (5.2%)	3 (1.9%)	2 (1.3%)	155
Visible symptoms, body image, and embodied distress	29 (46.8%)	20 (32.3%)	6 (9.7%)	2 (3.2%)	1 (1.6%)	0 (0.0%)	4 (6.5%)	62
Nutrition, weight, and metabolic concerns	55 (53.9%)	19 (18.6%)	6 (5.9%)	9 (8.8%)	7 (6.9%)	2 (2.0%)	4 (3.9%)	102
Disclosure dilemmas and family expectations	15 (62.5%)	1 (4.2%)	1 (4.2%)	0 (0.0%)	4 (16.7%)	1 (4.2%)	2 (8.3%)	24
Healthcare access and specialist navigation	41 (53.9%)	17 (22.4%)	11 (14.5%)	1 (1.3%)	4 (5.3%)	2 (2.6%)	0 (0.0%)	76
Psychosocial burden and coping difficulties	3 (60.0%)	1 (20.0%)	0 (0.0%)	1 (20.0%)	0 (0.0%)	0 (0.0%)	0 (0.0%)	5
Alternative/self-directed management attempts	9 (52.9%)	2 (11.8%)	0 (0.0%)	3 (17.6%)	1 (5.9%)	2 (11.8%)	0 (0.0%)	17
Total	**398**	**137**	**50**	**47**	**39**	**22**	**15**	**708**

Values are *n* (row %). Each peer response was linked to the dominant theme of its corresponding initial post and coded to one dominant response pattern using the dominant-communicative-function rule.

Bold values indicate the total number of peer responses in each response category and the overall total.

## 4 Discussion

This study mapped PCOS-related discussions in a Turkish online forum by analyzing 120 initial posts together with 708 linked peer responses. The main contribution is the combination of concern-level thematic analysis with response-level content analysis and concern-by-response cross-tabulation. Initial posts most often centered on fertility, treatment, diagnostic uncertainty, visible symptoms, and metabolic or lifestyle concerns, while peer responses were dominated by incomplete or uncertain advice, with biomedical guidance, navigation, lived experience, emotional support, alternative recommendations, and stigmatizing content appearing less frequently.

The most frequent concern in initial posts was fertility, pregnancy, and motherhood anxiety. This finding is consistent with the reproductive relevance of polycystic ovary syndrome, as ovulatory dysfunction and fertility concerns are central components of clinical assessment and management (2). However, our findings extend guideline-based understanding by showing how fertility concerns appeared in everyday digital discourse. Users did not only ask whether pregnancy was biologically possible; they also expressed fear, urgency, desire for motherhood, and concern about family expectations. This aligns with previous evidence showing that polycystic ovary syndrome can affect quality of life through infertility-related, emotional, menstrual, weight, hirsutism, and body image domains (5, 6). It is also consistent with lived-experience research indicating that the condition can affect identity, relationships, self-management, and future expectations (8). The contribution of the present study is that fertility anxiety emerged as the most frequent dominant theme in naturally occurring Turkish forum posts, suggesting that reproductive uncertainty may be one of the most visible reasons users seek peer input online.

Treatment and medication-related uncertainty was the second most frequent theme. Users asked about oral contraceptives, antiandrogens, antidepressants, isotretinoin, ovulation induction medications, and which specialty should manage specific symptoms. This finding is consistent with studies reporting delayed diagnosis, inadequate information, dissatisfaction with care, and unmet needs for disease-management support among people with polycystic ovary syndrome (7, 9). Gibson-Helm et al. (7) showed that delayed diagnosis and lack of information were associated with dissatisfaction, while Sydora et al. (9) identified patient-perceived gaps in diagnosis, follow-up, and multidisciplinary care. Similar to these studies, our findings suggest that users may experience care as fragmented, especially when reproductive, dermatological, metabolic, and psychological symptoms are managed separately. Our study adds a digital dimension by showing that users bring this fragmentation into forum spaces, asking whether gynecology, dermatology, endocrinology, psychiatry, or nutrition services are most appropriate for their concerns.

Diagnostic uncertainty was also prominent. Users repeatedly asked about hormone test timing, ultrasound findings, and the meaning of having normal laboratory results despite persistent symptoms. This finding supports previous work showing that polycystic ovary syndrome diagnosis can be experienced as delayed, confusing, or insufficiently explained (7–9). It also aligns with Gomula et al. (10), who found that women used online health resources and social media to make sense of a diagnosis they perceived as complex and poorly understood. The present study extends this finding by showing the specific forms of diagnostic uncertainty expressed in forum posts: users asked when to test hormones, whether menstruation timing mattered, why ultrasound and blood test findings might not align, and whether symptoms were still valid when laboratory results were described as normal. This suggests that clinical communication should include practical explanations of diagnostic criteria and test interpretation, rather than assuming that a diagnostic label alone is sufficient.

Visible symptoms, body image, and embodied distress were central to users' narratives. Acne, oily skin, hirsutism, hair loss, body size, weight gain, and inability to gain weight were not described merely as cosmetic concerns; they were linked to shame, self-confidence, social participation, and daily life. This is consistent with the 2023 international guideline, which emphasizes the importance of psychological well-being and quality of life in polycystic ovary syndrome care (2). It also aligns with Lee and Dokras (6), who noted that body weight, hirsutism, acne, and negative body image are implicated in psychological distress among women with the condition. Current recommendations further emphasize the need to assess mental health symptoms, body image distress, eating-related concerns, psychosexual function, and quality of life (2, 4). Our study adds two points to this literature. First, visible symptoms were often a reason for online help-seeking, especially when users felt that laboratory results did not justify treatment. Second, several users described themselves as thin or unable to gain weight, challenging the common association between polycystic ovary syndrome and overweight. This suggests that patient education and online health communication should represent the heterogeneity of body size and symptom profiles more clearly.

Nutrition, weight, and metabolic concerns were also common. This finding is consistent with international recommendations that emphasize lifestyle and metabolic assessment as part of care (2). However, the forum data showed that lifestyle concerns were not limited to weight loss. Users also discussed inability to gain weight, sweet cravings despite normal blood sugar, gluten-free diets, food affordability, and fear of disease progression. These findings add nuance to clinical discussions of lifestyle management by showing that dietary advice is interpreted through feasibility, cost, and everyday constraints. If lifestyle recommendations are perceived as unrealistic or unaffordable, users may turn to peer advice or self-directed strategies, which can be helpful in some cases but may also increase exposure to incomplete or non-evidence-based recommendations.

Disclosure dilemmas and family expectations appeared less frequently but were analytically important. Users worried about whether to disclose the diagnosis to a partner, fiancé, spouse, or partner's family because the condition might be interpreted as infertility. Previous lived-experience research has shown that polycystic ovary syndrome may affect relationships, identity, and emotional well-being (8). Our findings contribute context-specific insight by showing how disclosure appeared as a relational decision in forum discussions. The concern was not only whether others would know about a diagnosis, but whether the diagnosis would be interpreted as reduced fertility or future reproductive difficulty. Because these findings come from one online forum, they should not be generalized to all Turkish users or all individuals with the condition. Nevertheless, they suggest that counseling may need to address not only fertility facts but also how patients understand, communicate, or choose not to disclose the diagnosis in intimate and family contexts.

The peer response analysis adds an important layer to previous research. Gomula et al. (10) showed that online resources and social media are used to understand polycystic ovary syndrome and find support, while Naroji et al. (11) demonstrated that polycystic ovary syndrome-related content is prevalent across TikTok, Instagram, and Reddit and may shape perceptions of the condition, trust in medical providers, and attitudes toward interventions. Our study extends this work by examining peer responses within linked forum threads. The results show that users did not only encounter static online content; they received responses that normalized symptoms, shared personal experiences, recommended services, minimized concerns, or suggested alternative remedies. This interactional perspective helps clarify how online communities may actively shape users' interpretation of symptoms, treatments, fertility, and care-seeking.

Incomplete or uncertain advice was the most frequent response pattern after revision-stage recoding (56.2%), followed by biomedical guidance or normalization (19.4%). This finding refines interpretations based solely on whether online content is accurate or inaccurate (11, 13, 14): many peer responses were not necessarily demonstrably false, but were too brief, unsupported, or decontextualized to resolve the user's question. The concern-by-response analysis further showed that this pattern cut across fertility, diagnostic, treatment, and healthcare-access discussions. In contrast, practical navigation was relatively more visible in healthcare-access threads, and biomedical guidance was relatively more common in discussions of visible symptoms. These relationships are descriptive and should not be interpreted as causal effects of forum participation.

Alternative or non-evidence-based advice represented a smaller proportion of peer responses (3.1%) but remained important because recommendations included herbal, supplement-based, and other self-directed approaches. This pattern is consistent with concerns raised in studies of PCOS-related online content (11, 13, 14). In the present forum, such advice often appeared alongside unresolved questions about fertility, medication, menstrual regulation, or dissatisfaction with care. The analysis therefore identifies where potential misinformation risk appears within peer interaction, but it does not establish that forum exposure changed users' subsequent health decisions.

Dismissive, stigmatizing, or inappropriate responses were uncommon overall (2.1%) but remained analytically important. They included minimization, body-related judgment, fertility-related mockery, and inappropriate or sexualized comments. Because visible symptoms, body image, and reproductive expectations can already contribute to psychological burden in PCOS (2, 4–6), even infrequent stigmatizing exchanges may be relevant to understanding the social context of online help-seeking. The present data describe the occurrence of these responses but cannot determine their effect on individual users.

### 4.1 Implications for practice

The findings have implications for women's health, reproductive health, dermatology, endocrinology, mental health, and nursing practice. Clinicians may benefit from recognizing that patients can arrive with questions shaped by online peer discussions, particularly about fertility, diagnostic testing, visible symptoms, medication, and alternative approaches. Clear explanations of diagnostic criteria, test timing, treatment options, and realistic fertility expectations may reduce the need to rely on fragmentary peer advice. Clinicians can also invite patients to discuss online information without assuming that forum use is either beneficial or harmful. The present study does not measure digital health literacy or demonstrate effects on health decisions; rather, it identifies recurring information needs and the types of peer responses available in one Turkish forum.

### 4.2 Strengths and limitations

This study has several strengths. It analyzed naturally occurring online discussions rather than researcher-elicited interviews and examined both initial concerns and the peer responses they elicited. The revision-stage re-audit strengthened the response-level analytic trail by distinguishing original-poster follow-up messages from peer responses and by applying explicit operational definitions across the response corpus. The concern-by-response cross-tabulation also adds a relational layer that is often absent from studies of static social-media content.

Several limitations should also be acknowledged. First, the data came from a single Turkish online forum and are subject to platform and search-selection bias; users who post publicly may differ systematically from people with PCOS who do not use such forums. Second, the platform workflow did not preserve a machine-readable count of all initial search-result hits, so the exact number of records identified and excluded before the final 120 eligible initial posts could not be retrospectively verified; this limits full reconstruction of the initial screening denominator. Third, demographic information was limited to visible, self-reported profile data, was missing for some users, and could not be verified; therefore, the sample cannot be treated as demographically representative of Turkish people with PCOS. Fourth, diagnoses, symptoms, and treatment histories were self-reported and could not be clinically verified. Fifth, non-verbatim researcher-generated summaries were used to reduce traceability, which may reduce linguistic nuance and interactional detail. Sixth, public forum text containing sensitive health narratives was processed within ChatGPT during AI-assisted organization and recoding; although no private platform data were collected and all outputs were researcher-reviewed, this constitutes an additional privacy and methodological consideration. Seventh, assigning one dominant theme to each initial post and one dominant function to each response necessarily simplifies multi-topic and multifunctional content. Finally, responses were not clinically fact-checked statement by statement, so the analysis describes response functions and potential misinformation risk rather than confirmed misinformation or downstream effects on health behavior.

## 5 Conclusion

PCOS-related discussions in this Turkish online forum were characterized by fertility anxiety, treatment and diagnostic uncertainty, visible-symptom and body-image concerns, metabolic and lifestyle questions, disclosure dilemmas, and healthcare-navigation needs. Across 708 peer responses, incomplete or uncertain advice was the most frequent response pattern, while biomedical guidance, practical navigation, lived experience, emotional support, alternative recommendations, and stigmatizing interactions occurred less often. Linking responses to the concerns that elicited them showed that different topics generated different mixes of peer input, although incomplete advice predominated across most categories. These findings describe one Turkish forum environment and should not be generalized to all people with PCOS/PMOS or assumed to demonstrate effects on health decisions. Future research should compare multiple Turkish digital platforms and prospectively examine how users interpret and act on peer advice.

## Data Availability

The raw data supporting the conclusions of this article will be made available by the authors, without undue reservation.
